# Circulating 4-F_4t_-Neuroprostane and 10-F_4t_-Neuroprostane Are Related to *MECP2* Gene Mutation and Natural History in Rett Syndrome

**DOI:** 10.3390/ijms22084240

**Published:** 2021-04-19

**Authors:** Cinzia Signorini, Silvia Leoncini, Thierry Durand, Jean-Marie Galano, Alexandre Guy, Valérie Bultel-Poncé, Camille Oger, Jetty Chung-Yung Lee, Lucia Ciccoli, Joussef Hayek, Claudio De Felice

**Affiliations:** 1 Department of Molecular and Developmental Medicine, University of Siena, 53100 Siena, Italy;ciccolil@yahoo.it; 2 Neonatal Intensive Care Unit, Azienda Ospedaliera Universitaria Senese, 53100 Siena, Italy;s.leoncini74@gmail.com; 3 Child Neuropsychiatry Unit, Azienda Ospedaliera Universitaria Senese, 53100 Siena, Italy;hayekjoussef@gmail.com; 4 Institut des Biomolécules Max Mousseron, (IBMM), UMR 5247, CNRS, Université de Montpellier, ENSCM, CEDEX 5, 34093 Montpellier, France; thierry.durand@umontpellier.fr (T.D.); jean-marie.galano@umontpellier.fr (J.-M.G.); alexandre.guy@umontpellier.fr (A.G.); valerie.bultel@umontpellier.fr (V.B.-P.); camille.oger@umontpellier.fr (C.O.); 5 School of Biological Sciences, The University of Hong Kong, Hong Kong; jettylee@hku.hk; 6 Pediatric Speciality Center “L’Isola di Bau”, 50052 Certaldo, Florence, Italy

**Keywords:** *MECP2* mutation, natural history, neurological disease, neuroprostanes, phenotype, Rett syndrome

## Abstract

Neuroprostanes, a family of non-enzymatic metabolites of the docosahexaenoic acid, have been suggested as potential biomarkers for neurological diseases. Objective biological markers are strongly needed in Rett syndrome (RTT), which is a progressive X-linked neurodevelopmental disorder that is mainly caused by mutations in the methyl-CpG binding protein 2 (*MECP2*) gene with a predominant multisystemic phenotype. The aim of the study is to assess a possible association between *MECP2* mutations or RTT disease progression and plasma levels of 4(*RS*)-4-F_4t_-neuroprostane (4-F_4t_-NeuroP) and 10(*RS*)-10-F_4t_-neuroprostane (10-F_4t_-NeuroP) in typical RTT patients with proven *MECP2* gene mutation. Clinical severity and disease progression were assessed using the Rett clinical severity scale (RCSS) in n = 77 RTT patients. The 4-F_4t_-NeuroP and 10-F_4t_-NeuroP molecules were totally synthesized and used to identify the contents of the plasma of the patients. Neuroprostane levels were related to *MECP2* mutation category (i.e., early truncating, gene deletion, late truncating, and missense), specific hotspot mutations (i.e., R106W, R133C, R168X, R255X, R270X, R294X, R306C, and T158M), and disease stage (II through IV). Circulating 4-F_4t_-NeuroP and 10-F_4t_-NeuroP were significantly related to (i) the type of *MECP2* mutations where higher levels were associated to gene deletions (*p* ≤ 0.001); (ii) severity of common hotspot *MECP2* mutation (large deletions, R168X, R255X, and R270X); (iii) disease stage, where higher concentrations were observed at stage II (*p* ≤ 0.002); and (iv) deficiency in walking (*p* ≤ 0.0003). This study indicates the biological significance of 4-F_4t_-NeuroP and 10-F_4t_-NeuroP as promising molecules to mark the disease progression and potentially gauge genotype–phenotype associations in RTT.

## 1. Introduction

Biomarkers are increasingly employed in empirical studies of human populations to understand how physiological processes change with the diseases [1,2,3,4,5]. The identification of biomarkers is strongly needed for Rett syndrome (RTT; MIM #312750), which is an X-linked neurodevelopmental disorder that affects most females [6]. It is mainly (>90%) caused by mutations in the *methyl CpG binding protein 2* (*MECP2*) gene (OMIM #300005) coding for MeCP2, which is a pleotropic protein abundantly expressed in the brain. MeCP2 is involved as an epigenetic modulator by controlling chromatin architecture and gene expression through the binding to methylated DNA [7] and acting as a key regulator for neuronal development and function [8,9]. Over nine hundred *MECP2* mutations have been reported including benign and pathogenic variations [10], with the most frequent ones (“hotspots”) comprising more than 3/4 (78%) of all the reported pathogenic mutations [11].

Main features include a severe stereotypic intellectual disability, seizures, autonomic dysfunction, microcephaly, communication dysfunction, postural hypotonia, growth failure, and non-purpose hand use [12]. The typical clinical picture of the disease is diagnosed as a peculiar phenotypic RTT form by a panel of key clinical elements [13] with a median age of diagnosis at 2.7 years (interquartile range: 2–4.1 years) [14]. The disease evolves by highlighting clinical changes, which are classified according to a sequence of disease stages. In particular, in typical RTT, a four-stage neurological regression occurs with loss of previously acquired cognitive, social, and motor skills. After 6–8 months of life with apparent normal psychomotor development, RTT children experience a phase of neurological regression during which they lose manual skills and speech and develop hand stereotypies, autistic behaviors, and walking deficiencies. Subsequently, these patients show a pseudo-stationary stage that is followed by a stage where further motor deterioration, characterized by scoliosis and worsening of the ability to walk, takes place [6,10].

In recent years, natural history (NH) [15,16], clinical severity [17,18], and genotype–phenotype association [17,19,20,21] have become relevant in better understanding the disease progression and developing objective measurements to be applied in trial metrics [22].

No definitive cure for RTT is available to date [23]. Several trials are ongoing in order to test molecules that are potentially able to improve quality of life [24], although no objective measures to assess clinical severity at the cellular, biochemical, or molecular level are currently available.

Cumulating evidence indicates a key role of oxidative stress in the pathogenesis of RTT [25,26]. Isoprostanoids, a large family of compounds derived from non-enzymatic oxidation of polyunsaturated fatty acids (PUFAs) [27,28], have been shown to be pertinent to RTT pathological mechanisms [26,29,30]. Among the isoprostanoids, neuroprostanes (NeuroPs), metabolites produced by oxidative metabolism of docosahexaenoic acid (DHA) in the neural cells, have been reported to be relevant in RTT and other conditions of neurological disease [30,31,32]. Prior data strongly suggest that in vivo DHA oxidation follows preferential chemical rearrangements according to different brain diseases [30]. Among the eight regioisomer series (4-, 7-, 10-, 11-, 13-, 14-, 17- or 20-series) generated by free radical-induced oxidation of DHA, 4(*RS*)-4-F_4t_-neuroprostane (4-F_4t_-NeuroP) and 10(*RS*)-10-F_4t_-neuroprostane (10-F_4t_-NeuroP) are the most investigated ones [11,29,30]. In particular, plasma levels of 4-F_4t_-NeuroP and 10-F_4t_-NeuroP have been suggested to be biologically synthesized in vivo and related to clinical severity in different neurological diseases in order to be distinctive for different neurological conditions [30].

The aim of the study is to assess a possible association between *MECP2* mutations or RTT disease progression and plasma levels of 4-F_4t_-NeuroP and 10-F_4t_-NeuroP in typical RTT patients harboring *MECP2* gene mutations.

## 2. Results

Gas chromatography/negative-ion chemical ionization tandem mass spectrometry (GC/NICI-MS/MS) was used to detect and quantify 10-F_4t_-NeuroP and 4-F_4t_-NeuroP in plasma samples from all the patients (range values: 1.0–15.75 pg/mL and 0.73–3.75 pg/mlL, respectively). Reference range values in matched control subjects (n = 45) were 0.0–2.1 pg/mL (median 0.0, interquartile range 0.0–0.35) and 0.0–0.9 pg/mL (median 0.0, interquartile range 0.0–0.3) for circulating 10-F_4t_-NeuroP and 4-F_4t_-NeuroP, respectively. RTT population and control subjects showed comparable age (*p* = 0.8502).

### 2.1. Relevance of 10-F_4t_-NeuroP and 4-F_4t_-NeuroP Plasma Levels to RTT Disease Stage

One of the primary endpoints of the study was the evaluation of the relationship between neuroprostane molecules and RTT disease stage. Clinically, the disease stage was evaluated in the examined RTT population. No stage I patients were present (Table 1).

**Figure 1:**
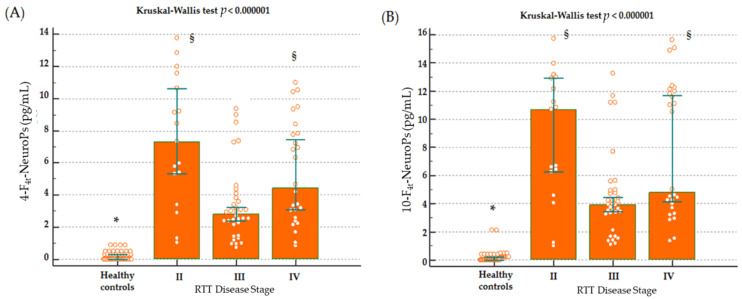
Plasma levels of 4-F_4t_-NeuroP (**A**) and 10-F_4t_-NeuroP (**B**) as a function of RTT disease stage. Data are shown as medians (columns) and interquartile ranges (25th–75th percentiles). RTT subjects: n = 77, Healthy controls n = 45. * different from stages II, III, and IV; § different from Stages III and healthy controls, *post-hoc* analysis, Conover test, *p* < 0.05.

Plasma levels of both 10-F_4t_-NeuroP and 4-F_4t_-NeuroP were significantly different in subjects at different stages of the disease (Figure 1). Circulating levels of 4-F_4t_-NeuroP and 10-F_4t_-NeuroP were significantly higher in stage II and stage IV as compared to stage III.

### 2.2. Relevance of 10-F_4t_-NeuroP and 4-F_4t_-NeuroP Plasma Levels to MECP2 Mutation Type and to MeCP2 Protein Domain

A further primary endpoint was the comparison between plasma levels of both 10- and 4-F_4t_-NeuroP, and *MECP2* mutation category.

In RTT subjects, *MECP2* mutation type was established (Table 2), and the distribution of *MECP2* mutation categories in each disease stage is displayed in Table 3. Moreover, although hundreds of unique mutations in *MECP2* have been identified to date, the eight most frequent ones (R106W, R133C, R168X, R255X, R270X, R294X, R306C, T158M) causing RTT, along with carboxy-terminal deletions (C-terminal deletions) and large deletions that are “hotspots” mutations, were found.

**Figure 2:**
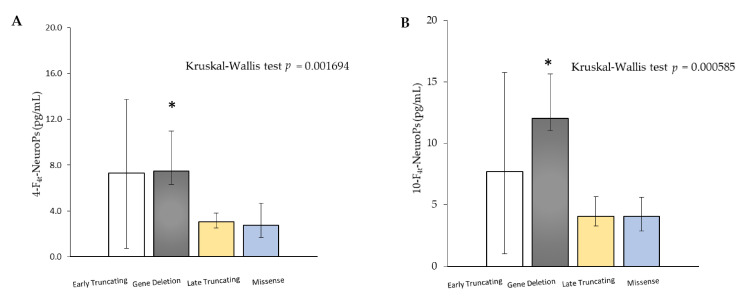
Plasma levels of 4-F_4t_-NeuroP (**A**) and 10-F_4t_-NeuroP (**B**) in RTT subjects as a function of *MECP2* mutation categories. Data are medians (columns) and bars are interquartile ranges (25th–75th percentiles). * Different from early truncating, late truncating, and missense mutations; *post hoc* analysis, Conover test *p* < 0.05.

Statistical multiple comparisons showed the amount of plasma for both 4-F_4t_-NeuroP and 10-F_4t_-NeuroP were significantly different in RTT population related to *MECP2* mutation categories (Figure 2).

**Figure 3:**
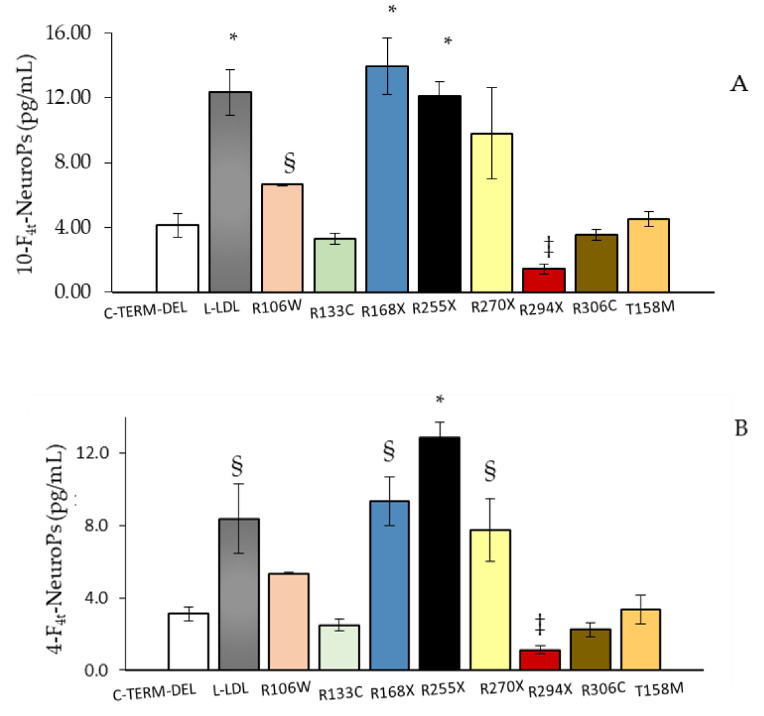
Plasma levels of 10-F_4t_-NeuroP (**A**) and 4-F_4t_-NeuroP (**B**) in RTT subjects as a function of *MECP2* “hotspots” mutation. Data are means (columns) with standard deviations. ANOVA test: *p* < 0.001 (**A**,**B**) and Scheffé test for all pairwise comparisons: *p* < 0.05. Panel A: * different from C-Terminal deletion, R106W, R133C, R294X, R306C, and T158M; ‡ different from C-TERM_DEL, L-DEL, R106W, R168X, R255X, R270X, R294X, T158M; § different from 270X: Panel B: * different from all reported mutations; § different from C-TERM-DEL, R133C, R255X, R294X, R306C, and T158M; ‡ different from all the hotspots mutations, with the exception of R133C and R306C. Legend: C-TERM-DEL, C-terminal deletions; L-DEL, large deletions.

In addition, plasma levels of both 10-F_4t_-NeuroP and 4-F_4t_-NeuroP were significantly different in RTT subjects carrying different “hotspots” mutations (Figure 3).

**Figure 4:**
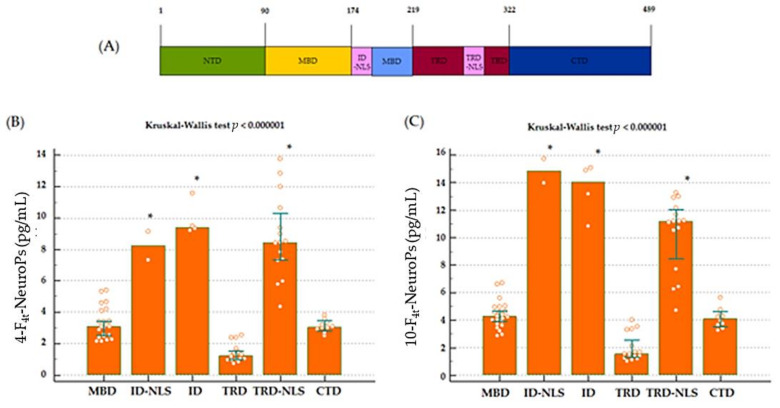
Plasma levels of 4-F_4t_-NeuroP and 10-F_4t_-NeuroP in RTT subjects with different affected MeCP2 protein domain. In panel (**A**) schematic representation of MeCP2 protein structure, amino acid number is reported. In panel (**B**,**C**) plasma levels of 4-F_4t_-NeuroP and 10-F_4t_-NeuroP in RTT patients with affected MeCP2 protein in different protein domain. None of the mutations in the investigated RTT subjects was located at the NTD protein domain Legend: NTD: N-terminal domain; MBD: methyl binding domain; ID: Inter domain; ID-NLS: Inter domain—nuclear localization signal; TRD: Transcriptional repression domain; TRD-NLS: Transcriptional repression domain—nuclear localization signal; CTD: C-terminal domain. Data are expressed as medians and interquartile ranges. * Different from CTD, MBD, and TRD, *post hoc* analysis (Conover), *p* < 0.05.

A deeper relationship between F_4t_-NeuroP molecules and *MECP2* gene was investigated by analyzing plasma levels of both 4-F_4t_-NeuroP and 10-F_4t_-NeuroP as a function of the MeCP2 protein domains affected by patients’ mutations. The enrolled subjects showed alteration in C-terminal domain (CTD, n = 10), inter domain (ID, n = 4), inter domain—nuclear localization signal (ID-NLS, n = 2), methyl binding domain (MBD, n = 22), transcriptional repression domain (TRD, n = 16), transcriptional repression domain—nuclear localization signal (TRD-NLS, n = 15), or in whole protein (n = 8). For both F_4t_-NeuroP isomers, multiple comparisons showed that neuroprostane plasma levels associated to alteration in ID-NLS, ID, TRD-NLS, or whole protein were significantly different from those associated to the other affected domain (Figure 4).

### 2.3. Relevance of 10-F_4t_-NeuroP and 4-F_4t_-NeuroP Plasma Levels to RTT Clinical Severity

**Figure 5:**
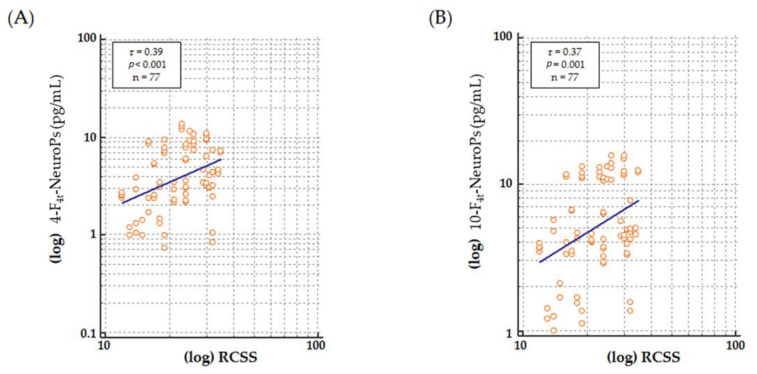
Scatterplot and linear regression of plasma 4-F_4t-_NeuroP (**A**) and 10-F_4t_-NeuroP (**B**) against. RTT clinical severity (RCSS). In each panel, correlation coefficient (r), *p* value (*p*), and number of patients (n) are displayed.

It is known that specific mutations in *MECP2* confer different severity in RTT [17,20]; therefore, in addition to the relationship between plasma neuroprostane levels and *MECP2* mutation (Figure 2, Figure 3 and Figure 4), the distribution of circulating neuroprostane molecules as a function of clinical severity was evaluated. After logarithmic transformation, significant, although moderate, positive relationships were observed between disease clinical severity (RCSS) and 4-F_4t-_NeuroP or 10-F_4t_-NeuroP plasma levels (Figure 5).

Patients’ NH and clinical severity scale were estimated by Rett clinical severity scale (RCSS) by evaluating the thirteen item RCSS (Table 4).

Four out of the thirteen-item RCSS (as reported in Table 4) showed significant positive relationships with 4- or 10-F_4t_-NeuroP levels. In particular, walking deficiency was the RCSS item more strongly related to F_4t_-NeuroP levels (Table 5 and Table 6).

## 3. Discussion

Our findings show that 4-F_4t_-NeuroP or 10-F_4t_-NeuroP, previously reported to be relevant to neurological diseases [29,30,31], are specifically linked to RTT severity, natural history, and *MECP2* mutation type.

No cure for RTT is currently available, although a number of treatments either downstream [24] or gene-targeted (i.e., gene therapy, gene editing, and gene re-expression) [23] are proposed. In order to test the effects of potential treatments, the availability of objective and reliable biomarkers is of paramount relevance. The existing severity scores mainly suffer from subjectivity and lack of real-time monitoring. In recent years, biosensors based on artificial intelligence, machine learning, and internet of things have been proposed to overcome the intrinsic limitations of the subjective assessment as outcome measure in randomized clinical trials [33] (https://clinicaltrials.gov/ct2/show/NCT04514549, accessed date 18 April 2021).

On the other hand, reliable biomarkers should be able to guide diagnosis, prevention, severity monitoring, and follow-up, as well as assessment of treatment efficacy.

Although lipid peroxidation cannot be considered as a universal surrogate for oxidative stress evaluation [34], consistent evidence indicates that the levels of lipid peroxidation products are valuable biomarkers in measuring oxidative stress status [29,35]. The amounts of 4-F_4t_-NeuroP or 10-F_4t_-NeuroP in brain tissue have been reported to be predictive of severity score in a murine model of Krabbe disease [31] and symptomatic *Mecp*2 stop/y mice [30]. Moreover, in the *Mecp2*−/y mouse model of RTT, significant inverse relationships between F_4_-NeuroPs and brain weight were reported, thus suggesting the involvement of DHA-derived peroxidation products in the pathogenesis of microcephaly [26], which is a key clinical feature of the RTT phenotype. Interestingly, in the present study, a significant relationship between F_4t_-NeuroP (both 4-F_4t_-NeuroP and 10-F_4t_-NeuroP isomers) and microcephaly is observed in the examined RTT patient cohort.

Here, we investigated the role of F_4t_-NeuroPs over a broad spectrum of RTT phenotypes, while previous studies investigated the role of F_4_-NeuroPs in RTT [30,36]. The novelty of the current study implements specific isomers of F_4_-NeuroPs, namely 4-F_4t_-NeuroP and 10-F_4t_-NeuroP, in the pathogenetic mechanisms of RTT that contribute to the presentation and evolution of the disease. The F_4t_-NeuroP precursor i.e., DHA, is particularly abundant in the membranes of neurons as compared to other polyunsaturated fatty acids which are widely distributed in human tissues. DHA autoxidizes non-enzymatically and releases predominantly F_4t_-NeuroPs. In addition, the free radical-induced oxidation of PUFAs has been consistently reported in RTT patients [37,38,39] as well as in the brain tissue of *Mecp2* mutant experimental mice [26]. Given that RTT is a genetic disease linked to impaired expression of a regulatory gene of genetic transcription and chromatin structure, this rare neurodevelopmental disease is considered a useful model in the comprehension of common mechanisms shared by neurodegenerative diseases. It is important to underline that although clinical severity typically worsens, RTT is considered a neurodevelopmental disorder [40].

*MECP2* mutation type is a strong predictor of disease severity [12,17,41]. Interestingly, plasma amounts of both 4-F_4t_-NeuroP and 10-F_4t_-NeuroP showed different abundance as a function of *MECP2* gene mutation type. According to our results, showing higher amounts of F_4_-NeuroP isomers in subjects carrying either early truncating mutations or gene deletions, a more severe disease is reported to be closely related to *MECP2* truncating mutations [17,20,42,43,44]. Furthermore, 4-F_4t_-NeuroP and 10-F_4t_-NeuroP plasma levels mirrored the clinical severity known in patients carrying *MECP2* “hotspots” mutations [17]. Therefore, F_4_-NeuroP isomers could be involved in the impact of different *MECP2* mutations on ambulation, hand use, and language deficiencies [17]. Interestingly, plasma levels of both 4-F_4t_-NeuroP and 10-F_4t_-NeuroP are shown to be significantly different also considering the MeCP2 protein domain along which gene mutations are grouped. Moreover, mutations after the NLS MeCP2 protein domain (mutations including R294 onwards through C terminal) were found to be associated to a significant decrease in severity of motor functions [45] and are, in our study, associated to lower plasma levels of both 4- and 10-F_4t_-NeuroPs.

Since the aim here is to identify the relationship between F_4_-NeuroP isomers and RTT genotype–phenotype correlation, or NH of RTT, a unique RTT population with proven *MECP2* mutations was assessed, and an intra-group comparison was performed. Likewise, a comparative study was carried out to investigate if specific mutations in *MECP2* confer different severity in RTT [17]. Moreover, significant increase in both 4-F_4t_-NeuroP and 10-F_4t_-NeuroP in RTT subjects as compared to a matched control population was previously reported [30]. A similar study, involving the RTT genotype–phenotype and, in particular NH in RTT, would be difficult to investigate in mouse RTT models given the different temporal evolution of the disease in mice compared to humans. [26].

Higher levels of both 4-F_4t_-NeuroP and 10-F_4t_-NeuroP at stage II of the disease, is worthy of debate, as it reinforces the link between the investigated F_4_-NeuroP isomers and the biological mechanisms leading to disease progression. The so defined stage II represents a period of rapid-developmental regression due to the loss of previously acquired skills such as communication and gross motor skills. Conversely, in stage III, the phenotype stabilizes, and some of the skills lost during stage II may be also be partially regained. Stage IV is the last disease stage where the late motor deterioration can last for years or decades [10]. Thus, the time-course for 4-F_4t_-NeuroP and 10-F_4t_-NeuroP formation appears to be parallel to the neurological impairment resulting in the clinical presentation. One further point to be taken into account in the study is that no subjects at stage I were enrolled, since it is the early onset stagnation period, which appears between 6 and 18 months of age, and it is frequently overstepped at the time of the clinical diagnosis.

The wide clinical spectrum of RTT appears to be linked to a variety of *MECP2* gene targets [12,46]. Under the clinical consensus criteria [13], key clinical features are used for the distinction between classic or typical RTT from variant or atypical forms [43]. To date, the role of possible co-existing home mutations in influencing oxidative stress in RTT is unclear [47], and no biochemical indicators of neurological disease progression in the natural history of RTT have been identified. More research is needed to understand the relationship between oxidized products of DHA and data coming from exome sequencing.

Although more insight is certainly needed on the mechanistic side, overall, our data indicate that 4-F_4t_-NeuroP and 10-F_4t_-NeuroP are involved in the pathophysiological mechanisms of RTT and mark the patient’s NH.

## 4. Materials and Methods

### 4.1. Subjects

A total of 77 typical RTT patients (age: range: 5.0–47.0; median 15.0; 95% C.I. for the median 13.0 to 19.0, interquartile range 10.0–23.0) with proven hotspot *MECP2* gene mutation (R294X, n = 12. R133C, n = 6, R306C, n = 4, C-term deletion, n = 10, R106W, n = 2; T158M, n = 14, R270X, n = 12, R255 X, n = 3, R168X, n = 6; Large deletion, n = 8) admitted to the Child Neuropsychiatry Unit of the Azienda Ospedaliera Universitaria Senese (Siena, Italy) from Jan 2012 to June 2014 (Head: J.H.). Moreover, a total of 45 matched for gender and of comparable age (age: range: 5.00–45.0; median 15.0; 95% C.I. for the median 15.0 to 17.0, inter-quartile range 13.0–18.0) healthy control subjects were recruited. Written consent form was obtained by all the enrolled subjects or by the patient’s guardians. All sensitive clinical data were anonymized by assigning a randomly generated integer code to each RTT patients/control subjects. This study was approved by the local Ethical Committee of Siena University Hospital (Azienda Ospedaliera Universitaria Senese, Siena, Italy)and was carried out in accordance to the rules expressed in the Declaration of Helsinki Ethical Principles for Medical Research involving Human Subjects (Brazil, 2013). A structured clinical evaluation was carried out for each patient.

### 4.2. RTT Natural History and Clinical Severity Scoring

RTT natural history (NH) was recorded through longitudinal data, and the following key variables were considered: scoliosis, muscle tone, sitting, ambulation, hand function, and feeding.

Clinical severity was assessed by Rett Clinical Severity Score (RCSS) [17]. RCSS is a validated RTT specific scale designed to assess the severity of key symptoms was completed by the clinicians (C.D.F. and J.H.) at the periodic medical check-ups. RCSS consists of 13 items (age of onset of regression, somatic growth, head growth, independent sitting, ambulation (independent or assisted), hand use, scoliosis, language, non-verbal communication, respiratory dysfunction, autonomic symptoms, onset of stereotypies, and seizures), providing a rating of core symptoms of RTT on a Likert scale of either 0 to 4 or 0 to 5 with a maximum total score of 58. All scores range from 0 to 4 or 0 to 5 with 0 representing the least severe and 4 or 5 representing the most severe finding.

### 4.3. Sample Preparation

Platelet-poor plasma samples were obtained by centrifugation (2400× *g* for 15 min at 4 °C) of blood aliquots collected in heparinized tubes. As an antioxidant, butylated hydroxytoluene (BHT) (90 μM prepared in absolute ethanol) was added to each plasma samples, mixed and stored at −70 °C until the time of detection and quantification of F_4t_-NeuroPs (4-F_4t_-NeuroP and 10-F_4t_-NeuroP) released into the circulation as unesterified F_4t_-NeuroPs (free F_4_-NeuroPs).

### 4.4. 4(RS)-F4t-NeuroP, and 10(R)-10-F4t-NeuroP and 10(S)-10-F4t-NeuroP Synthesis

**Scheme 1:**
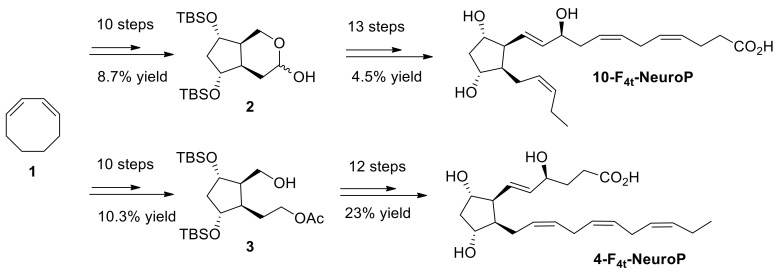
Steps of the synthesis of 4- and 10-F_4t_-NeuroPs. Details are described in the text.

Both 4- and 10-F_4t_-NeuroP molecules are not commercially available for quantitative analysis in targeted lipidomics. Hence, it is in-house synthesized for the use in this study. The synthesis of the two series of 4- and 10-F_4t_-NeuroPs was performed by our group in previous work [48,49,50], as summarized in Scheme 1. The two compounds were obtained in around 20–22 steps of synthesis from commercially available 1,3-cyclooctadiene 1. As an example of the synthetic work, Scheme 1 describes the synthesis of the 4- and the 10-F_4t_-NeuroP.

Following our recent strategy [48], the two key bicyclic intermediates 2 and 3 were obtained in 10 steps and in 8.7% and 10.3% yield, respectively. The introduction of α and ω chains was performed by using regioselective protections/deprotections, oxidations, Wittig elongation, and cross metathesis coupling reactions as the main steps for the 10-F_4t_-NeuroP [50]. The final step was the saponification of the methyl esters in the presence of LiOH to obtain free acids. The 4-F_4t_-NeuroP was obtained starting from intermediate 3 in 12 more steps of synthesis, giving 23% yield after optimizations, while 10-F_4t_-NeuroP and its C10-epimer were obtained in 13 steps from intermediate 2 [49].

### 4.5. 4-F_4t_-NeuroP and 10-F_4t_-NeuroP Measurement

Measurement of F_4_-NeuroPs (4-F_4t_-NeuroP and 10-F_4t_-NeuroP) was performed by a gas chromatography/negative-ion chemical ionization tandem mass spectrometry (GC/NICI-MS/MS) after sequential extraction and derivatization steps.

For purification of circulating F_4_-NeuroPs, a series of clean-up procedures were carried out using known methods [30,51]. Briefly, the internal standard (PGF_2α_-d_4_, 500 pg in 50 μL ethanol) was added to each plasma sample (1 mL), together with a volume of 2 mL acidified water (pH 3), and then two sequential extractions on solid phase (SPE), the first on a C_18_ cartridge and the second on an NH_2_ cartridge, were performed. The C_18_ cartridge (500 mg Sorbent per Cartridge, 55–105 µm Particle Size, 6cc, Waters, Milford, MA, USA) was preconditioned with methanol (5 mL) and water (5 mL), and sequentially washed after loading the sample with 10 mL water (pH 3), and 10 mL water: acetonitrile (85:15, *v*/*v*). Hexane: ethyl acetate: propan-2-ol (30:65:5 *v*/*v*/*v*, 5 mL) mix was used for the final eluate. Consecutively, the eluate obtained from C_18_ cartridge was transferred to the NH_2_ cartridge (500 mg Sorbent per Cartridge, 55–105 µm Particle Size, 6cc, Waters, Milford, MA, USA), which was preconditioned with hexane (5 mL). After loading the eluate, sequential wash with 10 mL of hexane: ethyl acetate (30:70, *v*/*v*), 10 mL acetonitrile: water (9:1, *v*/*v*), and 10 mL acetonitrile were performed, until the final elution carried out with a mix of ethyl acetate: methanol: acetic acid (10:85:5, *v*/*v*/*v*, 5 mL). The eluate collected from the NH_2_ cartridge was evaporated under nitrogen at 40 °C before carrying out the derivation step.

In the derivatization process, the carboxylic group of the F_4_-NeuroPs, as well as for PGF_2α_-d_4_, was converted into pentafluorobenzyl ester, while the hydroxyl group was converted to trimethylsilyl ethers. To this end, an incubation for 45 min at 40 °C was carried out in the presence of 40 μL of pentafluorobenzyl bromide (10% in acetonitrile) and 20 μL of diisopropylethylamine (10% in acetonitrile). Subsequently, the solvent was evaporated under a stream of nitrogen, and 50 μL of N,O-bis(trimethylsilyl)trifluoroacetamide with 5 μL of diisopropylethylamine (10% in acetonitrile) were added for a second incubation at 45 °C for 1 h. One more time, the samples were dried and re-suspended in 50 μL of undecane containing bis(trimethylsilyl)trifluoroacetamide (10%) for GC/NICI-MS/MS analysis (SPB 1701 GC capillary column 30 m × 0.25 mm i.d., 0.25 μm film thickness; helium as the carrier gas at 1 mL/min flow rate; methane as reagent gas, at 2.0 mL/min flow rate). The mass ions determined were the product ions at *m/z* 323 and *m/z* 303 derived from the [M-181]^−^ precursor ions of F_4_-NeuroPs (*m/z* 593) and PGF_2α_-d_4_ (*m/z* 573) respectively, which corresponds to the loss of CH_2_C_6_F_5_ from the derivatization process. Quantification of F_4_-NeuroPs was performed by relating the analyte/internal standard peak area ratio (F_4t_-NeuroP/PGF_2α_-d_4_) to the calibration curve constructed.

### 4.6. Data Analysis

Differences between groups were evaluated by multiple comparisons carried out by Kruskal–Wallis test and post hoc analysis (Conover) or by one-way analysis of variance (ANOVA). The associations between variables were tested using the Spearman rank correlation at 95% confidence intervals (95% C.I.). A two-tailed *p* < 0.05 was considered to indicate statistical significance. Data were analyzed by using the MedCalc ver. 12.0 statistical software package (MedCalc. Software, Mariakerke, Belgium).

## 5. Conclusions

This study indicates that circulating 4-F_4t_-NeuroP and 10-F_4t_-NeuroP isomers, non-enzymatic oxidized products of DHA, are objective biomarkers for neurological severity in RTT. Plasma levels of these molecules mirror key symptoms of the disease. Remarkably, circulating levels of the molecules are correlated to RCSS neurological items and *MECP2* mutation type, thus indicating that lipid peroxidation in the brain gray matter (concentrated with DHA) is involved in shaping the individual NH in RTT patients.

## Figures and Tables

**Table 1:** Disease stage of the examined RTT population.

Examined Patients (N)	77
Clinical Stage *	Subjects in Each Category (N)
Stage II	17
Stage III	34
Stage IV	26

* A typical disease stage is referred as a symptomatic phase of the disease where a specific cluster of phenotypical symptoms appear during the disorder progression [10].

**Table 2:** *MECP2* mutations of the examined RTT population.

Examined Patients (N)	77
*MECP2* Mutation Category	Subjects in Each Category (N)
Early truncating	35
Gene deletion	8
Late truncating	10
Missense	24

**Table 3:** Distribution of *MECP2* mutation categories for each RTT disease stage.

*MECP2* Mutation Category	*MECP2* Mutation Category in Each Disease Stage (N)		
	Stage II	Stage III	Stage IV
Early truncating	15	14	6
Gene deletion	0	0	8
Late truncating	0	8	2
Missense	2	12	10

**Table 4:** Rett Clinical Severity Scale (RCSS) in RTT examined population.

Parameters Evaluated in Examined Patients	Median	C.I.	Value Range
RCSS	24.0	21 to 24.9	7–44
RCSS sub-items			
- Regression onset	3	2–3	0–4
- Stereotype onset	3	2–3	0–4
- Microcephaly	2	2–3	0–4
- Somatic growth deficiency	1	1–2	0–4
- Sitting impairment	0	0–0	0–5
- Walking deficit	1	1–1	0–5
- Purposeful hand use deficit	3	3–3	0–4
- Scoliosis	1	1–1.9	0–5
- Verbal language deficiency	3	3–3	0–4
- Non-verbal communication deficiency	0	0–0	0–3
- Respiratory dysfunction	2	2–2	0–4
- Autonomic nervous system dysfunction	1	1–1	0–4
- Seizures	1	1–1	0–4

Legend C.I., confidence interval.

**Table 5:** Correlation matrix for 4-F_4t_-NeuroP as a function of RCSS sub-components.

4-F_4t_-NeuroP Correlation Parameters	Walking Deficit	Verbal Language Deficiency	Deficit in Purposeful Hand Use	Microcephaly	Seizures
Spearman’s coefficient *Rho*	0.438	0.261	0.321	0.264	0.251
*p* value	**0.0001**	**0.0221**	**0.0045**	**0.0203**	**0.0279**
95% C.I. for *Rho*	0.237 to 0.603	0.0388 to 0.458	0.104 to 0.508	0.0425 to 0.461	0.0283 to 0.449

Legend: C.I., confidence interval. Significant associations are highlighted in bold. Subjects: n = 77.

**Table 6:** Correlation matrix for 10-F_4t_-NeuroP as a function of RCSS sub-components.

10-F_4t_-NeuroP Correlation Parameters	Walking Deficit	Verbal Language Deficiency	Deficit in Purposeful Hand Use	Microcephaly	Seizures
Spearman’s coefficient *Rho*	0.404	0.289	0.284	0.235	0.276
*p* value	**0.0003**	**0.0108**	**0.0123**	**0.04**	**0.0150**
95% C.I. for *Rho*	0.198 to 0.576	0.0696 to 0.482	0.0640 to 0.478	0.0112 to 0.436	0.0557 to 0. 471

Legend: C.I., confidence interval. Significant associations are highlighted in bold. Subjects: n = 77.

## Data Availability

Data are available in the individual medical record of each patient at Child Neuropsichiatry Unit, Azienda Ospedaliera Universitaria Senese (Siena, Italy).

## Abbreviations

4-F_4t_-NeuroP	4(RS)-4-F_4t_-neuroprostane
10-F_4t_-NeuroP	10(RS)-10-F_4t_-neuroprostane
BMI	body mass index
DHA	docosahexaenoic acid
F_4_-NeuroPs	F_4_-neuroprostanes
GC/NICI-MS/MS	gas chromatography/negative-ion chemical ionization tandem mass spectrometry
PUFA	polyunsaturated fatty acid
RTT	Rett syndrome
RCSS	Rett clinical severity scale
NH	natural history

## References

[1] Mayeux R. (2004). Biomarkers: Potential uses and limitations. NeuroRx.

[2] Crimmins E., Vasunilashorn S., Kim J.K., Alley D. (2008). Biomarkers related to aging in human populations. Adv. Clin. Chem..

[3] Guo X., Tan W., Wang C. (2020). The emerging roles of exosomal circRNAs in diseases. Clin. Transl. Oncol..

[4] Nassar S.F., Raddassi K., Ubhi B., Doktorski J., Abulaban A. (2020). Precision Medicine: Steps along the Road to Combat Human Cancer. Cells.

[5] d’Abramo C., D’Adamio L., Giliberto L. (2020). Significance of Blood and Cerebrospinal Fluid Biomarkers for Alzheimer’s Disease: Sensitivity, Specificity and Potential for Clinical Use. J. Pers. Med..

[6] Samaco R.C., Neul J.L. (2011). Complexities of Rett syndrome and MeCP_2_. J. Neurosci..

[7] Zachariah R.M., Rastegar M. (2012). Linking epigenetics to human disease and Rett syndrome: The emerging novel and challenging concepts in MeCP_2_ research. Neural Plast..

[8] Ehrhart F., Coort S.L., Cirillo E., Smeets E., Evelo C.T., Curfs L.M. (2016). Rett syndrome: Biological pathways leading from MECP_2_ to disorder phenotypes. Orphanet J. Rare Dis..

[9] Lyst M.J., Bird A. (2015). Rett syndrome: A complex disorder with simple roots. Nature Rev. Genet..

[10] Gold W.A., Krishnarajy R., Ellaway C., Christodoulou J. (2018). Rett syndrome: A genetic update and clinical review focusing on comorbidities. ACS Chem. Neurosci..

[11] Christodoulou J., Grimm A., Maher T., Bennetts B. (2003). RettBASE: The IRSA MECP2 variation database-a new mutation database in evolution. Hum. Mutat..

[12] Chahrour M., Zoghbi H.Y. (2007). The story of Rett syndrome: From clinic to neurobiology. Neuron.

[13] Hagberg B., Hanefeld F., Percy A., Skjeldal O. (2002). An update on clinically applicable diagnostic criteria in Rett syndrome. Comments to Rett Syndrome Clinical Criteria Consensus Panel Satellite to European Paediatric Neurology Society Meeting, Baden Baden, Germany, 11 September 2001. Eur. J. Paediatr. Neurol..

[14] Tarquinio D.C., Hou W., Neul J.L., Lane J.B., Barnes K.V., O’Leary H.M., Bruck N.M., Kaufmann W.E., Motil K.J., Glaze D.G. (2015). Age of diagnosis in Rett syndrome: Patterns of recognition among diagnosticians and risk factors for late diagnosis. Pediatr. Neurol..

[15] Kerr A.M., Stephenson J.B. (1986). A study of the natural history of Rett syndrome in 23 girls. Am. J. Med. Genet. Suppl..

[16] Nomura Y., Segawa M. (2005). Natural history of Rett syndrome. J. Child. Neurol..

[17] Neul J.L., Fang P., Barrish J., Lane J., Caeg E.B., Smith E.O., Zoghbi H., Percy A., Glaze D.G. (2008). Specific mutations in methyl- CpGbinding protein 2 confer different severity in Rett syndrome. Neurology.

[18] Kaufmann W.E., Tierney E., Rohde C.A., Suarez-Pedraza M.C., Clarke M.A., Salorio C.F., Bibat G., Bukelis I., Naram D., Lanham D.C. (2012). Social impairments in Rett syndrome: Characteristics and relationship with clinical severity. J. Intellect. Disabil. Res..

[19] Charman T., Neilson T.C., Mash V., Archer H., Gardiner M.T., Knudsen G.P., McDonnell A., Perry J., Whatley S.D., Bunyan D.J. (2005). Dimensional phenotypic analysis and functional categorisation of mutations reveal novel genotype-phenotype associations in Rett syndrome. Eur. J. Hum. Genet..

[20] Cuddapah V.A., Pillai R.B., Shekar K.V., Lane J.B., Motil K.J., Skinner S.A., Tarquinio D.C., Glaze D.G., McGwin G., Kaufmann W.E. (2014). Methyl-CpG-binding protein 2 (MECP_2_) mutation type is associated with disease severity in Rett syndrome. J. Med. Genet..

[21] Fabio R.A., Colombo B., Russo S., Cogliati F., Masciadri M., Foglia S., Antonietti A., Tavian D. (2014). Recent insights into genotype-phenotype relationships in patients with Rett syndrome using a fine grain scale. Res. Dev. Disabil..

[22] Oberman L.M., Downs J., Cianfaglione R., Leonard H., Kaufmann W.E. (2020). Assessment of a Clinical Trial Metric for Rett Syndrome: Critical Analysis of the Rett Syndrome Behaviour Questionnaire. Pediatr. Neurol..

[23] Sandweiss A.J., Brandt V.L., Zoghbi H.Y. (2020). Advances in understanding of Rett syndrome and MECP2 duplication syndrome: Prospects for future therapies. Lancet Neurol..

[24] Vashi N., Justice M.J. (2019). Treating Rett syndrome: From mouse models to human therapies. Mamm. Genome.

[25] De Felice C., Ciccoli L., Leoncini S., Signorini C., Rossi M., Vannuccini L., Guazzi G., Latini G., Comporti M., Valacchi G. (2009). Systemic oxidative stress in classic Rett syndrome. Free Radic. Biol. Med..

[26] De Felice C., Della Ragione F., Signorini C., Leoncini S., Pecorelli A., Ciccoli L., Scalabrì F., Marracino F., Madonna M., Belmonte G. (2014). Oxidative brain damage in Mecp_2_-mutant murine models of Rett syndrome. Neurobiol. Dis..

[27] Galano J.M., Lee Y.Y., Oger C., Vigor C., Vercauteren J., Durand T., Giera M., Lee J.C. (2017). Isoprostanes, neuroprostanes and phytoprostanes: An overview of 25 years of research in chemistry and biology. Prog. Lipid Res..

[28] Leung K.S., Galano J.M., Durand T., Lee J.C. (2015). Current development in non-enzymatic lipid peroxidation products, isoprostanoids and isofuranoids, in novel biological samples. Free Radic. Res..

[29] Signorini C., De Felice C., Galano J.M., Oger C., Leoncini S., Cortelazzo A., Ciccoli L., Durand T., Hayek J., Lee J.C. (2018). Isoprostanoids in Clinical and Experimental Neurological Disease Models. Antioxidants.

[30] Signorini C., De Felice C., Durand T., Galano J.M., Oger C., Leoncini S., Ciccoli L., Carone M., Ulivelli M., Manna C. (2018). Relevance of 4-F4t-neuroprostane and 10-F4t-neuroprostane to neurological diseases. Free Radic. Biol. Med..

[31] Signorini C., Cardile V., Pannuzzo G., Graziano A.C.E., Durand T., Galano J.M., Oger C., Leoncini S., Cortelazzo A., Lee J.C. (2019). Increased isoprostanoid levels in brain from murine model of Krabbe disease—Relevance of isoprostanes, dihomo-isoprostanes and neuroprostanes to disease severity. Free Radic. Biol. Med..

[32] Miller E., Morel A., Saso L., Saluk J. (2014). Isoprostanes and neuroprostanes as biomarkers of oxidative stress in neurodegenerative diseases. Oxid. Med. Cell Longev..

[33] Kadhim K.T., Alsahlany A.M., Wadi S.M., Kadhum H.T. (2020). An Overview of Patient’s Health Status Monitoring System Based on Internet of Things (IoT). Wirel. Pers. Commun..

[34] Dotan Y., Lichtenberg D., Pinchuk I. (2004). Lipid peroxidation cannot be used as a universal criterion of oxidative stress. Prog. Lipid Res..

[35] Santos R., Almodovar C.R., Bulteau A.L., Gomes C.M. (2013). Neurodegeneration, neurogenesis, and oxidative stress. Oxidative Med. Cell. Longev..

[36] Signorini C., De Felice C., Leoncini S., Giardini A., D’Esposito M., Filosa S., Della Ragione F., Rossi M., Pecorelli A., Valacchi G. (2011). F₄-neuroprostanes mediate neurological severity in Rett syndrome. Clin. Chim. Acta..

[37] Signorini C., De Felice C., Leoncini S., Durand T., Galano J.M., Cortelazzo A., Zollo G., Guerranti R., Gonnelli S., Caffarelli C. (2014). Altered erythrocyte membrane fatty acid profile in typical Rett syndrome: Effects of omega-3 polyunsaturated fatty acid supplementation. Prostaglandins Leukot Essent Fatty Acids.

[38] Signorini C., De Felice C., Leoncini S., Møller R.S., Zollo G., Buoni S., Cortelazzo A., Guerranti R., Durand T., Ciccoli L. (2016). MECP2 Duplication Syndrome: Evidence of Enhanced Oxidative Stress. A Comparison with Rett Syndrome. PLoS ONE.

[39] De Felice C., Signorini C., Leoncini S., Pecorelli A., Durand T., Valacchi G., Ciccoli L., Hayek J. (2012). The role of oxidative stress in Rett syndrome: An overview. Ann. N. Y. Acad. Sci..

[40] Neul J.L., Zoghbi H.Y. (2004). Rett syndrome: A prototypical neurodevelopmental disorder. Neuroscientist.

[41] Maortua H., Martínez-Bouzas C., García-Ribes A., Martínez M.J., Guillen E., Domingo M.R., Calvo M.T., Guitart M., Gabau E., Botella M.P. (2013). MECP_2_ gene study in a large cohort: Testing of 240 female patients and 861 healthy controls (519 females and 342 males). J. Mol. Diagn..

[42] Cheadle J.P., Gill H., Fleming N., Maynard J., Kerr A., Leonard H., Krawczak M., Cooper D.N., Lynch S., Thomas N. (2000). Long-read sequence analysis of the MECP2 gene in Rett syndrome patients: Correlation of disease severity with mutation type and location. Hum. Mol. Genet..

[43] Neul J.L., Kaufmann W.E., Glaze D.G., Christodoulou J., Clarke A.J., Bahi-Buisson N., Leonard H., Bailey M.E., Schanen N.C., Zappella M. (2010). Rett syndrome: Revised diagnostic criteria and nomenclature. Ann. Neurol..

[44] Caffarelli C., Gonnelli S., Pitinca M.D.T., Camarri S., Al Refaie A., Hayek J., Nuti R. (2020). Methyl-CpG-binding protein 2 (MECP_2_) mutation type is associated with bone disease severity in Rett syndrome. BMC Med. Genet..

[45] Shovlin S., Tropea D. (2018). Transcriptome level analysis in Rett syndrome using human samples from different tissues. Orphanet J. Rare Dis..

[46] Lasalle J.M., Yasui D.H. (2009). Evolving role of MeCP_2_ in Rett syndrome and autism. Epigenomics.

[47] Grillo E., Lo Rizzo C., Bianciardi L., Bizzarri V., Baldassarri M., Spiga O., Furini S., De Felice C., Signorini C., Leoncini S. (2013). Revealing the complexity of a monogenic disease: Rett syndrome exome sequencing. PLoS ONE.

[48] Oger C., Brinkmann Y., Bouazzaoui S., Durand T., Galano J.M. (2008). Stereocontrolled access to isoprostanes via a bicyclo[3.3.0]octene framework. Org. Lett..

[49] Oger C., Bultel-Poncé V., Guy A., Balas L., Rossi J.C., Durand T., Galano J.M. (2010). The handy use of Brown’s catalyst for a skipped diyne deuteration: Application to the synthesis of a d4-labeled-F4t-neuroprostane. Chem. Eur. J..

[50] Guy A., Oger C., Hepekauzen J., Signorini C., Durand T., De Felice C., Fürstner A., Galano J.M. (2014). Oxygenated metabolites of n-3 polyunsaturated fatty acid as potential oxidative stress biomarkers: Total synthesis of 8-F3t-IsoP, 10-F4t-NeuroP, and [D4]−10-F4t-NeuroP. Chem. Eur. J..

[51] Nourooz-Zadeh J., Liu E.H., Anggård E., Halliwell B. (1998). F4-isoprostanes: A novel class of prostanoids formed during peroxidation of docosahexaenoic acid (DHA). Biochem. Biophys Res. Commun..

